# CDKN2A is a prognostic biomarker and correlated with immune infiltrates in hepatocellular carcinoma

**DOI:** 10.1042/BSR20211103

**Published:** 2021-10-06

**Authors:** Jun-peng Luo, Jing Wang, Jin-hua Huang

**Affiliations:** 1 Department of Minimally Invasive Interventional Therapy, Sun Yat-sen University Cancer Center, State Key Laboratory of Oncology in South China, Collaborative Innovation Cancer for Cancer Medicine, 651 Dongfeng Road East, Guangzhou 510060, Guangdong Province, People’s Republic of China; 2 Department of General Medicine, the First Medical Center, Department of Chinese PLA General Hospital, Beijing, China

**Keywords:** CDKN2A, Hepatocellular carcinoma, Immune signature, Prognostic biomarker, Survival, Tumor-infiltrating

## Abstract

Cyclin dependent kinase inhibitor 2A (CDKN2A) is an essential regulator of immune cell functionality, but the mechanisms whereby it drives immune infiltration in hepatocellular carcinoma (HCC) remain unclear. In the present study, we studied the association with CDKN2A expression and immune invasion with the risk of developing HCC. A totally of 2207 different genes were found between HCC and adjacent liver tissues from TCGA and GEO databases. CDKN2A was highly expressed in HCC and associated with poorer overall survival and disease-free survival. Notably, CDKN2A expression was positively correlated with infiltrating levels into purity, B cell, CD+8 T cell, CD+4 T cell, macrophage, neutrophil, and dendritic cells in HCC. CDKN2A expression showed strong correlations between diverse immune marker sets in HCC. These findings suggest that CDKN2A expression potentially contributes to regulation of tumor-associated macrophages and can be used as a prognostic biomarker for determining prognosis and immune infiltration in HCC.

## Introduction

Liver cancer is considered to be the sixth major prevalent malignancy and the fourth cause of cancer-associated deaths worldwide. The 5-year survival rate of patients is usually less than 20% [1,2]. Hepatocellular carcinoma (HCC) is the main histological subtype of liver cancer, accounting for 80% of primary liver cancer. About 70% of patients with early-stage liver cancer relapsed into 5 years after surgery or radiofrequency ablation [3–5]. Tumor stag and risk stratification are important for the treatment of HCC. However, the molecular mechanisms underlying tumor formation and progression are poorly understood, which further complicates the effective treatment of HCC. In addition, the lack of markers that are specific to tumor type or disease stage indicates a critical gap in the current understanding and treatment of HCC.

CDKN2A encodes the P16 gene involved in a series of cellular pathways, including promoting tumor cell proliferation, inhibiting tumor cell apoptosis, inducing tumor stromal angiogenesis and reducing cancer cell sensitivity to chemotherapy [6]. Recent studies have shown that CDKN2A gene is associated with poor prognosis in a variety of cancers, such as pancreatic cancer, bladder cancer, and pancreatic ductal adenocarcinoma [7–9]. Previous studies have found that immune cells are widely distributed over tumor microenvironment. Shi et al. [10] found that the number of PD-1+, CD8+, T cells in tumor or circulation was positively correlated with the progression and recurrence of HCC. Immune infiltration is pivotal for the parthenogenesis of cancers [11,12]. The infiltration of leukocytes around the tumor vessels was also proved to be an independent risk factor of the prognosis of HCC [13]. Nowadays, the role of abnormal expression of tumor immune-related genome in the process of tumor immune escape has become a new direction of tumor research [14,15]. Many studies have shown that abnormal expression of immune genome plays an important role in the prognosis of patients with non-small cell lung cancer, ovarian cancer, gastric cancer, and renal cell carcinoma [16–19]. Recently, studies have shown that the phenomenon of immune infiltration may provide a new perspective on the treatment of liver cancer, and CDKN2A is related to the immune infiltration of HCC [20–22]. It is urgent to explore the potential molecular mechanism of HCC and explore new prognostic-related molecular markers for its clinical diagnosis and treatment.

In the present study, we comprehensively analyzed the expression of CDKN2A and its correlation between prognostic value of HCC via different software, including Oncomine, PrognoScan, and Kaplan–Meier plotter. The relationship between CDKN2A and the degree of immune infiltration was analyzed by TIMER and GEPIA database. Subsequently, CDKN2A protein level was individually assessed in HCC tissues. In the present study, we observed that CDKN2A highly expressed across HCC and it may affect the prognosis of patients by interacting with infiltrating immune cells.

## Materials and methods

### Download of TCGA and GEO datasets

The transcriptome gene expression profiles and clinical information of HCC were obtained from The Cancer Genome Atlas (https://xena.ucsc.edu/) and Gene Expression Omnibus (GEO, https://www.ncbi.nlm.nih.gov/geo/) [23]. The TCGA dataset included 373 HCC tissues and 50 adjacent tissues samples. Data Release 27.0 - October 29, 2020. Meanwhile, a total of 10 liver cancer datasets of *Homo sapiens* were downloaded from the official website of GEO, including GSE57957, GSE14520, GSE22058, GSE46444, GSE54236, GSE36376, GSE64041, GSE76297, GSE76427, and GSE102079 [24].

### Oncomine database analysis

CDKN2A mRNA expression levels in different cancer types were compared with their matched para cancer tissues using Oncomine (https://www.oncomine.org/) analysis in our experiment. The parameters were setting as follows: *P*-value < 0.001, |Fold change| > 2.

### GEPIA database analysis

The GEPIA database (http://gepia.cancer-pku.cn/index.html) [25] was used to verify the relevant results obtained from the application of the Oncomine database, and then “Survival Plots” module were applied to analyze the survival prognosis of CDKN2A. Moreover, the “Correlation Analysis” module from GEPIA explored the relationship between the expression of the CDKN2A gene and the immune gene markers.

### TIMER database analysis

The correlation between CDKN2A gene expression and a large number of immune infiltrating cells in HCC was analyzed by the gene module of The Tumor Immune Estimation Resource (TIMER, https://cistrome.shinyapps.io/timer/) [26]. In this research, we utilized “Gene” module to estimate the correlation between CDKN2A expression and immune infiltration level. Moreover, we selected the immune gene markers by searching the website of CellMarker (http://biocc.hrbmu.edu.cn/CellMarker/). The expression scatterplots can visualize correlations between CDKN2A and each immune gene marker.

### Analyses of CDKN2A expression and clinical phenotypes

CDKN2A expression levels among different tumor stages (TNM stage) were assessed by *t*-test and ANOVA analyses. To assess the relationship of CDKN2A expression of overall survival, the median of CDKN2A expression of each tumor was used as cutoff values to divide patients into two groups, and Cox proportional hazards models were employed. Cox proportional hazards model was used to generate hazard ratio (HR) and 95% confidence interval (CI) for each cancer types. Kaplan Meier plotter was used to analyze the relationship between CDKN2A expression and survival in HCC.

### Immunohistochemical staining of clinical tissue

A total of 35 HCC patients undergoing hepatectomy between 2015 and 2020 in the First Medical Center Department of Chinese PLA General Hospital were included in the present study. Immunohistology was performed on 4-mm paraffin-embedded formalin-fixed biopsies sections using anti-CDKN2A (Abcam 108349) antibodies. The antigen retrieval was obtained at 97°C in a citrate buffer with a pH of 6. The revelation system was based on an one-step biotin-free immunoperoxydase stain (Envision, DAKO, Glostrup, Denmark) using 3,3-diamino-benzidine chromogen (DAKO, Glostrup, Denmark) substrate followed by Hemalun counterstaining. Negative controls for each slide were processed concurrently with probed samples by omitting primary antibody.

### Statistical analysis

The differential genes from TCGA or GEO database were analyzed by R-packet (“limma”) [27]. Correlation data sets about the CDKN2A expression of cancer and adjacent tissues were created in Oncomine with P-values, fold changes, and gene ranks. Survival curves were drawn by the PrognoScan, Kaplan Meier plotter, and GEPIA. The hazard ratio and Cox *P*-values or log rank *P*-values was used for comparing OS and RFS among patients in different groups. The correlation between gene expression was analyzed in GEPIA and TIMER, in which Spearman’s correlation was employed as correlation coefficient. *P* values < 0.05 was considered statistically significant.

## Results

### High expressions of CDKN2A gene in hepatocellular carcinoma

**Figure 1:**
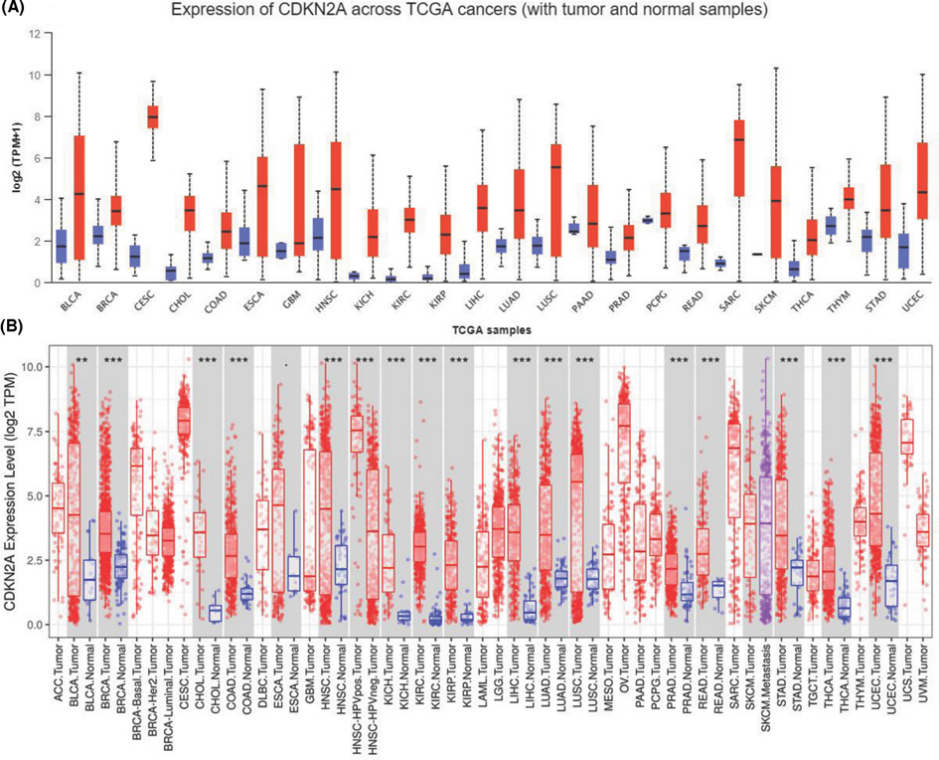
The mRNA expression of CDKN2A in various normal tissues and tumors (**A**) Increased or decreased expression of CDKN2A in different cancer tissues, compared with normal tissues in TCGA databases (Data Release 27.0 - October 29, 2020). (**B**) CDKN2A is differentially expressed between tumor and normal tissues in some cancers from TCGA and GEO databases. Each boxplot represents CDKN2A expression [RNA-seq RSEM, log2(normalized count +1)] across different cancers. Red is for tumors and blue is for normal tissues. The gray box shows that the expression of CDKN2A in liver cancer tissues is significantly higher than that in adjacent normal tissues. *P*-value significant codes: 0 ≤ *** < 0.001 ≤ ** < 0.01 ≤ * < 0.05 ≤ · < 0.1.

**Figure 2:**
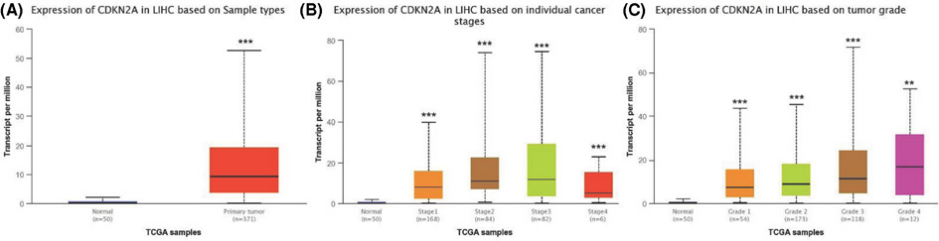
The mRNA expression of CDKN2A in various normal tissues and tumors (**A**) The expression levels of CDKN2A members in TCGA hepatocellular carcinoma dataset. (**B** and **C**) Box plot of relationship between CDKN2A expression level and clinical stage or grades of tumor. *P*-value significant codes: ***, *P*<0.001, **, *P*<0.01.

To comprehensively analyze CDKN2A expression and distribution in human normal tissues and tumor tissues, we analyzed CDKN2A mRNA expression levels in liver cancer datasets from GEO and 33 different tumor tissues from Xena (https://xenabrowser.net/). The expression of CDKN2A was highly variable across different normal tissues and tumor tissues (Figure 1A,B). Similarly, the detailed results of CDKN2A expression across different cancer types are summarized in UALCAN database (Figure 2A). To further verify the expression levels in cancerous and normal tissues of CDKN2A across all TCGA tumors, the data showed that the expression of CDKN2A was significantly increased to different stages and grades of cancer (Figure 2B,C).

### CDKN2A was a potential immune marker for HCC

**Figure 3:**
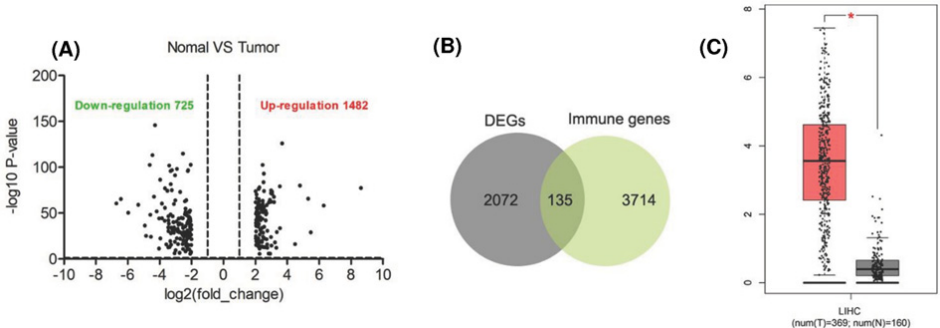
Procedure for the selection and validation of the prognosis biomarkers in hepatocellular carcinoma (**A**) Volcano plot of changes in gene expression in normal and tumor tissue. The latest data release of TCGA (Data Release 27.0 - October 29, 2020). Plots are the crosses of *P* values on the *y* axes and the ratio of gene expression on the *x* axes. The cutoff criteria (|log fold change| ≥ 1; *P*≤0.05) are indicated. (**B**) Venn diagram comparing DEGs in normal and tumor tissue with immune genes. Overlapping numbers represent the number of mutual genes between treatments. (**C**) The expression levels of CDKN2A members in GEO dataset. *P*-value significant code: *, *P*<0.05.

The expression data onto HCC were downloaded from TCGA database, including 50 control groups and 373 cancer tissues. Among them, 1482 genes were up-regulated and 725 genes were down-regulated in HCC tissues (Figure 3A). A total of 135 differentially expressed genes were screened by cross-comparison of 2072 differentially expressed genes with 3714 immune-related genes from Immport (https://www.immport.org/home) (Figure 3B). CDKN2A has the strongest correlation between immunity in 135 differentially expressed genes. What’s more, Similar to the results of GEO database, CDKN2A was highly expressed in tumor tissues and lowly expressed in normal tissues in TCGA database (Figure 3C).

### CDKN2A expressions correlated with prognosis in HCC

In the data on to 371 patients with liver cancer downloaded by TCGA, the patients were divided into CDKN2A high expression (*N*=232) group and CDKN2A low expression (*N*=139) group with the median expression of CDKN2A mRNA as the segmentation point (Clinical database sorting from TCGA database). The relationship of demographic and clinicopathological parameters with CDKN2A expression was analyzed using SPSS analysis. The results showed that CDKN2A expression was related to survival state (*P*=0.024) and TNM stage (*P*=0.032), but other parameters (sex, age, and grade) showed no correlation between CDKN2A expression (Table 1).

**Table 1:** Correlation between CDKN2A expression of clinicopathological from hepatocellular carcinoma patients

Clinicopathological factor	Number of cases	CDKN2A		χ^2^	*P*
		High expression	Low expression		
**Age**					
<55	117	68	49	0.546	
≥55	254	164	90		0.428
**Gender**					
Male	121	76	45	0.653	
Female	250	156	94		0.483
**TNM stage**					
I	158	103	55	6.735	
II	86	55	31		
III	127	74	53		**0.032**
**Grade**					
I	56	32	24	5.675	
II	177	104	73		
III	124	84	40		
IV	14	12	2		0.056
Survival status					
Death	126	97	29	6.974	
**Survival**	245	135	110		**0.024**

Data from TCGA database.

**Figure 4:**
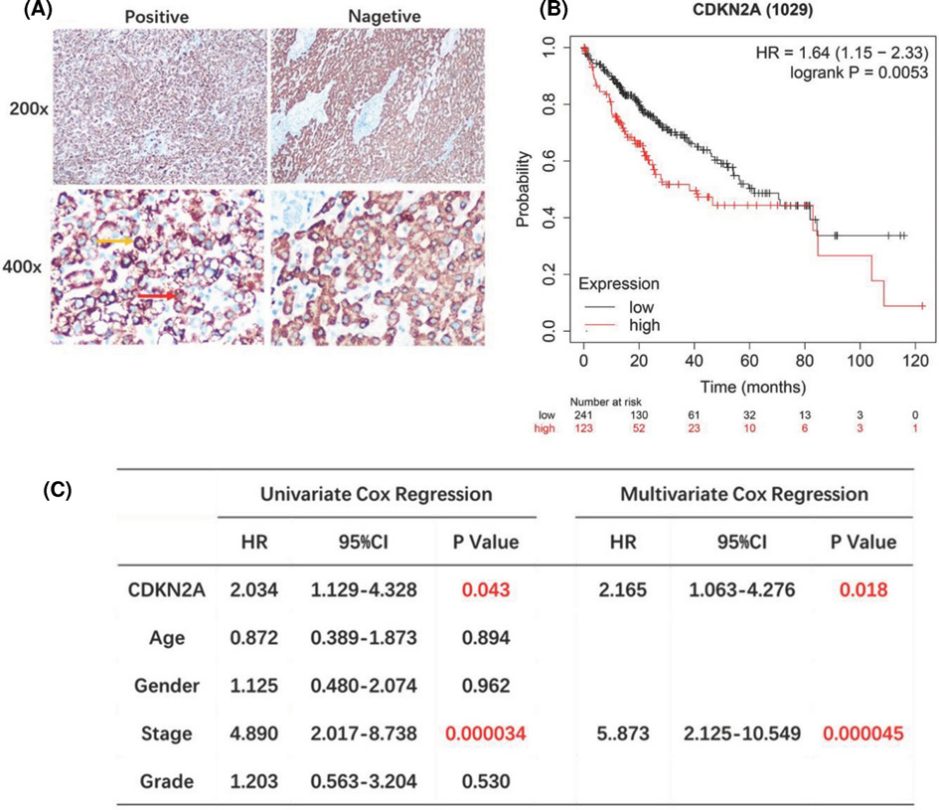
CDKN2A was independent prognostic factor of patients with hepatocellular carcinoma (**A**) Representative images of positive and negative CDKN2A protein expression of hepatocellular carcinoma tissues. CDKN2A was expressed in cytoplasm (yellow arrow) and nucleus (red arrow) of tumor cells. There were 35 patients stained positive and 35 patients stained negative. The scales bar to indicate 20 μm. (**B**) Kaplan–Meier analysis of CDKN2A protein in hepatocellular carcinoma patients. Patients with higher CDKN2A expression had shorter OS compared with patients with lower CDKN2A expression (*P*=0.0053, HR = 1.64). (**C**) Univariate and multivariate cox regression showed that higher CDKN2A protein expression and advanced pathological stage were independent prognostic factor in hepatocellular carcinoma patients. black: low mRNA expression.

**Figure 5:**
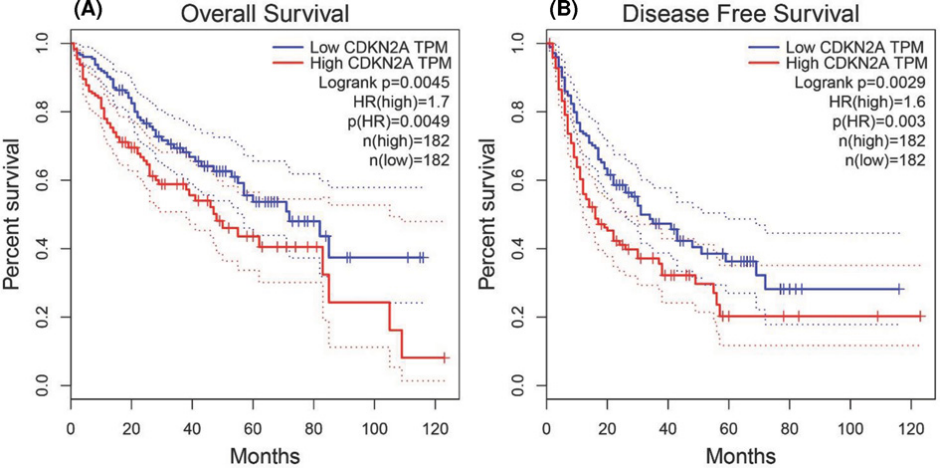
Prognostic values of CDKN2A in HCC Survival curves of OS (**A**) and DFS (**B**) comparing the high and low expression of CDKN2A in HCC from TCGA data in TIMER. Red: high mRNA expression; blue: low mRNA expression.

In addition, the expression of CDKN2A protein was explored for clinical tissue samples (35 cases of clinical liver cancer tissues and normal adjacent tissues were collected from the First Medical Center Department of Chinese PLA General Hospital) to validate the role of CDKN2A in hepatocellular carcinoma. Immunohistochemical results showed that the expression of CDKN2A was significantly higher than that in adjacent tissues (Figure 4A). Kaplan–Meier curves and log-rank test analyses confirmed that patients with positive CDKN2A expression had significantly shorter overall survival (OS) than patients with negative CDKN2A expression (*P*=0.0053, Figure 4B). Demographic and clinicopathological parameters of patients with hepatocellular carcinoma show in Table 2. Furthermore, cox proportional-hazards model was used to validate the potential for CDKN2A as a prognostic factor of hepatocellular carcinoma clinical tissue samples. Univariate cox regression suggested that CDKN2A expression (HR = 2.034, 95% CI: 1.129–4.328, *P*=0.043) and tumor stage (HR = 4.890, 95% CI: 2.017–8.738, *P*<0.001) were related to OS. Multivariate cox regression indicated that CDKN2A expression (HR = 2.165, 95% CI: 1.063–4.276, *P*=0.018) and tumor stage (HR = 5.873, 95% CI: 2.125–10.549, *P*<0.001) were independent prognostic factors for OS (Figure 4C). Moreover, Kaplan–Meier database was used to draw the survival curve between CDKN2A gene and the prognosis of HCC in TIMER. It was found that the expression of CDKN2A mRNA was associated with overall survival rate (OS) (HR = 1.7, *P*=0.0049) and disease-free survival (DFS) (HR = 1.6, *P*=0.003) (Figure 5A,B). In summary, CDKN2A was an independent prognostic factor of patients with hepatocellular carcinoma.

**Table 2:** Demographic and clinicopathological parameters of patients with hepatocellular carcinoma (data from 35 clinical patients)

Clinicopathological factor	Number of cases (*n*=35)	CDKN2A		*P*
		High expression	Low expression	
**Age**				
<55	7	5	2	
≥55	28	19	9	0.156
**Sex**				
Male	20	14	6	
Female	15	10	5	0.892
**TNM stage**				
I-II	16	11	5	0.053
III-IV	19	13	6	
Grade				
I-II	14	10	4	
III-IV	21	14	7	0.684
**Survival status**				
Death	12	10	2	
Survival	23	14	9	0.035

### CDKN2A expressions correlated with immune cell infiltration in Hepatocellular Carcinoma

**Figure 6:**
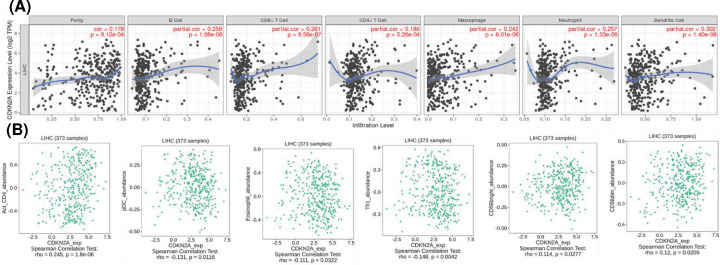
Correlation between CDKN2A expression of immune infiltration levels in hepatocellular carcinoma (**A**) CDKN2A expression has significant positive correlation between tumor purity, B cell, CD+8 Tcell, CD+4 Tcell, macrophage, neutrophil, and dendritic cell. *P*<0.05 is considered as significant. (**B**) Correlation analysis of CDKN2A and immune invasion related genes CD4, pDC, Eosinophil, Th1, CD56bright, and CD56dim in liver hepatocellular carcinoma. The *x*-axis represents the TPM of the CDKN2A gene (log2), and the *y*-axis represents the TPM of the genes CD4, pDC, Eosinophil, Th1, CD56bright, and CD56dim; TPM, transcript per million. *P*<0.05 is considered as significant.

Spearman’s correlation coefficient was applied to analyze the correlation between CDKN2A and immune infiltration level in TIMER. The analysis revealed that CDKN2A was positively correlated with tumor purity (cor = 0.178, *P*=0.000), B cells (cor = 0.259,* P*=0.0000), CD8 + T cells (cor = 0.261, *P*=0.000), neutrophils (cor = 0.257, *P*=0.000), especially with CD4 + T cells (cor = 0.186, *P*=0.000), macrophages (cor = 0.242, *P*=0.000) and dendritic cells (cor = 0.302, *P*=0.000) (Figure 6A). These results strongly suggested that CDKN2A played a specific role in the level of tumor cell immune infiltration in HCC.

### Association for CDKN2A with immune cells markers

In addition to the correlation between CDKN2A and the above six immune infiltrating cells, we next sought to find out whether CDKN2A was associated with the expression of more immune infiltrating cells by investigating related immune cell markers for HCC in TIMER and GEPIA. The results showed that the expression level of CDKN2A was significantly correlated with the immune marker genes of various immune cells in HCC (Figure 6B). Study observed that the expression of CDKN2A was strongly associated with 6 gene markers for 28 immune cell markers for HCC. Specifically, CD4 and marker genes of macrophages was significantly correlated with CDKN2A expression (*P*<0.00001). CD4+ T cells, macrophages and dendritic cells were most closely related to CDKN2A expression in HCC, which illustrated that CDKN2A expression of HCC associates with different degree of immune cell infiltration in different ways, further supporting that CDKN2A might be an effective factor influencing patients’ survival and prognosis.

## Discussion

There are few researches have reported CDKN2A in cervical cancer tissues was negatively correlated with serosal invasion [28]. And CDKN2A has been reported to promote the angiogenic phenotype and predict poor prognosis in esophageal squamous cell carcinoma [29]. In the present study, we systematically analyzed the expression of CDKN2A (mainly mRNA) in tumor tissues of HCC patients in TCGA and GEO databases. CDKN2A was highly expressed in cancer tissues and significantly impacted the prognosis of diverse cancers. What’s more, high CDKN2A expression was associated with poorer overall survival and disease-free survival. Almost all of the tumor samples and genomic and molecular data obtained by TCGA come from a single slice of the primary tumor of a newly diagnosed patient. The resulting genomic and molecular data cannot capture the tumor heterogeneity representing another patient’s outcome variable in any aspect of space or time [30]. Therefore, the expression levels of CDKN2A in 35 cases of HCC and adjacent normal tissues were also analyzed. It is worth noting that both clinical samples and TCGA database show that high expression of CDKN2A protein is usually associated with worse prognosis. The results shows that the expression of CDKN2A in HCC was significantly higher than that in normal tissues, and it was related to the prognosis of HCC.

Many evidences show that inflammation and immune dysfunction play an important role in the occurrence and development of HCC [31,32]. In the past few years, immunocheckpoint inhibitors and adoptive cell infusion have achieved good results in tumor immunotherapy, and have effectively improved the prognosis of HCC patients [33]. Recent studies conducted on cervical cancer suggested that patients who possess the CDKN2A methylation gene, as well as the drop in CDKN2A expression, suffered from a decreased OS rate [34]. The high expression of CDKN2A can promote the proliferation of cancer cells, inhibit the apoptosis of cancer cells, induce tumor interstitial angiogenesis, reduce the sensitivity of cancer cells to chemoradiotherapy, and ultimately affect the prognosis of HCC patients [35]. Recently, it has been reported that CDKN2A deletion can inhibit T-cell infiltration by inhibiting the expression of chemokines in a cell cycle dependent manner [36]. Although the specific molecular mechanism of CDKN2A regulation is still unclear, which may be related to p53 protein, CDKN2A is expected to become a target of HCC treatment in view of its important role in the occurrence and development of HCC.

The up-regulation of CDKN2A expression of HCC may be related to the involvement in CDKN2A in MAPK signaling pathway and HCC diversity [37]. In the present study, we systematically explored the differentially expressed genes related to HCC immunity. Similarly, the specific molecular mechanism of these immune-related genes in the occurrence and development of HCC was worthy to be studied further. The level of tumor immune infiltration will lead to the damage to the immune microenvironment and result from immune escape [38]. Many papers and reviews to suggest that multiple types of immune cells are associated with prognosis in various cancer types. In the present study, we observed that the high expression of CDKN2A was closely related to the decrease of OS in HCC patients, which has not be reported on previous studies. Therefore, we focused on whether CDKN2A expression is related to the level of immune invasion in HCC.

One important aspect of the present study is that we focused on the relationship between risk score and immune invasion in order to reveal its potential clinical significance. The expression of CDKN2A was correlated with various immune infiltration levels in HCC. Our results indicate that there is a positive correlation between the expression of CDKN2A and the immune infiltration of B cells, CD 8 + T cells, CD 4 + T cells, macrophages, neutrophils and dendritic cells, and the mutation of CDKN2A gene will affect the level of immune infiltrating cells in HCC. Moreover, the correlation between the expression of CDKN2A and immune cell marker genes suggests the role of CDKN2A in regulating tumor immunology of HCC. Specially, there is a strong correlation between CD4 + T cell gene marker (CD4) and CDKN2A expression. These results explain the potential role of CDKN2A in tumor-associated macrophage polarization.

In conclusion, the high expression of CDKN2A is associated with poor prognosis and decreased immune infiltration in HCC. In addition, CDKN2A expression may contribute to the regulation of tumor-associated macrophages, dendritic cells, and T cells. Therefore, CDKN2A may play an important role in immune infiltrating cells and be used as a prognostic biomarker for HCC patients. The present study provides a basis of the future research on the pathogenesis and treatment of HCC, and provides a new idea about the basic experiment of HCC and the development of new drugs.

## Data Availability

The data used to support the findings of this study are available from the corresponding author upon request.

## Abbreviations

CDKN2A: cyclin dependent kinase inhibitor 2A
DEG: different expression genes
DF: disease-free survival
GEPIA: Gene Expression Profiling Interactive Analysis
HCC: hepatocellular carcinoma
OS: overall survival
TCGA: The Cancer Genome Atlas
TIMER: tumor immune estimation resource
WHO: World Health Organization
