# Correction: CDKN2A is a prognostic biomarker and correlated with immune infiltrates in hepatocellular carcinoma

**DOI:** 10.1042/BSR-2021-1103_COR

**Published:** 2022-01-06

**Keywords:** CDKN2A, Hepatocellular carcinoma, Immune signature, Prognostic biomarker, Survival

The authors of the original article “CDKN2A is a prognostic biomarker and correlated with immune infiltrates in hepatocellular carcinoma.” (*Biosci Rep* (2021) **41**(10), https://doi.org/10.1042/BSR20211103) would like to correct Table 2. Due to their negligence, they had switched the number of high CDKN2A expression deaths with that of survivals in Table 2. This reversal in figures is corrected in this Correction.

**Table 2:** Demographic and clinicopathological parameters of patients with hepatocellular carcinoma (data from 35 clinical patients)

Clinicopathological factor	Number of cases(n = 35)	CDKN2A		*P*
		High expression	Low expression	
**Age**				
<55	7	5	2	0.156
≥55	28	19	9	
**Gender**				
Male	20	14	6	0.892
Female	15	10	5	
**TNM stage**				
I-II	16	11	5	0.053
III-IV	19	13	6	
**Grade**				
I-II	14	10	4	0.684
III-IV	21	14	7	
**Survival status**				
Death	16	14	2	**0.035**
Survival	19	10	9	

